# Biotechnological Applications and Health-Promoting Properties of Flavonols: An Updated View

**DOI:** 10.3390/ijms23031710

**Published:** 2022-02-01

**Authors:** Teresa Gervasi, Antonella Calderaro, Davide Barreca, Ester Tellone, Domenico Trombetta, Silvana Ficarra, Antonella Smeriglio, Giuseppina Mandalari, Giuseppe Gattuso

**Affiliations:** 1 Department of Biomedical and Dental Sciences and Morphofunctional Imaging, University of Messina, 98125 Messina, Italy; teresa.gervasi@unime.it; 2 Department of Chemical, Biological, Pharmaceutical and Environmental Sciences, University of Messina, V.le F. Stagno d’Alcontres 31, 98166 Messina, Italy; anto.calderaro@gmail.com (A.C.); ester.tellone@unime.it (E.T.); domenico.trombetta@unime.it (D.T.); silvana.ficarra@unime.it (S.F.); antonella.smeriglio@unime.it (A.S.); giuseppina.mandalari@unime.it (G.M.); giuseppe.gattuso@unime.it (G.G.)

**Keywords:** flavonols, phytonutrients, health-promoting properties, functional foods, biotechnological applications

## Abstract

Flavonols are a subclass of natural flavonoids characterized by a remarkable number of biotechnological applications and health-promoting properties. They attract researchers’ attention due to many epidemiological studies supporting their usage. They are phytochemicals commonly present in our diet, being ubiquitous in the plant kingdom and, in particular, relatively very abundant in fruits and vegetables. All these aspects make flavonols candidates of choice for the valorization of products, based on the presence of a remarkable number of different chemical structures, each one characterized by specific chemical features capable of influencing biological targets inside the living organisms in very different manners. In this review, we analyzed the biochemical and physiological characteristics of flavonols focalizing our attention on the most promising compounds to shed some light on their increasing utilization in biotechnological applications in processing industries, as well as their suitable employment to improve the overall wellness of the humankind.

## 1. Introduction

The use of natural compounds to increase the quality and shelf life of foods, especially as far as the production of functional foods is concerned, is a field in continuous development fueled by consumers’ increasing demand for natural products with health-promoting properties [1,2,3,4,5]. This increase is promoted by the contemporary development of ever more efficient extraction, isolation and identification techniques that allow producing nutraceuticals with high degree of purity at acceptable costs. One of the unique traits of flavonols is that, despite being low-molecular compounds, they display an astonishing variability in terms of structural features, so much so that, as of today, more than 300 individual derivatives have been reported in the literature. They are composed of a core moiety—often referred to as the aglycon—in which two aromatic rings (rings A and B) are connected via a pyran (or most commonly, a pyran-4-one) ring (ring C). This core moiety is generally decorated with a range of hydroxy and/or methoxy groups, as well as mono-, di- or oligosaccharides connected to the A ring or the B ring. Their biosynthesis takes place via the shikimic acid pathway. Phenylpyruvate is transformed into phenylalanine, which in turn is converted into trans-cinnamic acid by phenylalanine ammonia lyase, which is then oxidized to p-coumaric acid, a C-9 fragment. The latter is then condensed with three malonyl-CoA units to form a tetrahydroxychalcone. The closure of the pyran ring (ring C) leads to the formation of the flavonoid core [6]. The aim of the present review was to draw the attention of researchers and consumers to the biochemical and physiological characteristics of flavonols and, in particular, to the most promising compounds, analyzing and critically revising their biotechnological applications in processing industries, as well as their suitable employment for improving the overall wellness of the humankind, focusing our attention on the last 10–15 years. A few of the most common derivatives are shown in Table 1. The most representative—as well as the most common—flavonol is quercetin [7], a compound that has recently been indicated as an inhibitor of the 3Clpro protease of SARS-CoV-2 [8]. Quercetin, as the aglycon or its glycosylated derivatives, is also the most abundant flavonol (and one of the most abundant of all the flavonoid compounds) in human diet, totaling up to 40 mg/day [9]. This is because quercetin derivatives are very common in fruits and vegetables: onions, cauliflowers, broccoli, apples, tomatoes, grapes—all of them contain significant amounts that, taken together, contribute to regular intake for individuals with a healthy diet.

On top of those, berries [10], citrus fruits [11], spices [12] and beverages such as black or green tea also play an important role in dietary intake. Capers (*Capparis spinosa* L.) are, perhaps, the foodstuff with the highest quercetin content reported so far (234 mg/100 g, [13]. Kaempferol is the next most common, most abundant flavonol [14]. Capers contain 260 mg/100 g of kaempferol [13], the highest amount recorded in the literature, while saffron (obtained from the flowers of *Crocus sativus* L.) was shown to contain 205 mg/100 g [15]. Nonetheless, dietary intake of kaempferol comes mainly from many green leafy vegetables such as, for instance, arugula (*Eruca vesicaria* (L.) Cav.), Chinese cabbage (*Brassica rapa* L. var. Pekinensis), kale (*Brassica oleracea* L.), cress (*Lepidium sativum* L.) and watercress (*Nasturtium officinale*, W.T. Aiton), whose content varies between 15 and 60 mg/100 g, that are consumed in much larger amounts than the kaempferol-richer saffron or capers. Isorhamnetin has been the subject of fewer investigations than the better-known quercetin and kaempferol [16]. It has been found in high amounts in medicinal plants such as sea buckthorn (*Hippophae rhamnoides* L.) (up to 290 mg/100 g in its fruits) [17] or *Ginkgo biloba* (up to 120 mg/100 g in its leaves) [18]. Parsley (*Petroselinum crispum* (Mill.) Fuss) is also a rich source of isorhamnetin with a remarkable 330 mg/100 g dry weight [19]. It may also be worth mentioning the flavonol myricetin, which is present in significant amount in carob fibers, a dietary food supplement produced from carob (*Ceratonia siliqua* L.) fruit pulp (ca. 50 mg/100 g) [20]. However, the different distributions of flavonols and the occurrence in various dietary sources has been widely discussed elsewhere [21]. Details on the flavonol content of many other minor sources are available from public databases (http://www.ars.usda.gov/SP2UserFiles/Place/80400525/Data/Flav/Flav_R03-1.pdf, (accessed on 21 December 2021)).

## 2. Bioavailability and Metabolism of Flavonols

The term “bioavailability” means the fraction of a nutrient or non-nutrient that is available to the human body for physiological and/or storage functions. In other words, this term refers to the dose fraction that is available at the site of action. Normally, for doses administered orally, it refers to the dose fraction that reaches the systemic circulation. In the case of polyphenols, bioavailability involves different processes, such as (1) the solubility of polyphenols in the gastric and intestinal environments; (2) the release of polyphenols from the food matrix; (3) the degradation of polyphenols during the gastric and duodenal digestive processes; (4) the absorption of polyphenols by enterocytes; (5) phase I/II enzymatic modifications that occur after absorption; (6) transport into the systemic circulation and tissue distribution. Each of these steps plays a fundamental role in the process of absorption and metabolism of polyphenols [22]. The solubility of polyphenols represents a critical physicochemical parameter that shows a certain relationship with their bioavailability. However, this concept could be misleading because high solubility does not always correspond to high bioavailability and other parameters, such as the membrane permeability of enterocytes to polyphenols, must be taken into account. For this purpose, polyphenols can be classified into three categories: (1) high solubility but poor permeability through the cell membrane; (2) low solubility and poor permeability through the cell membrane; (3) low solubility and high permeability through the cell membrane. Many studies have shown that the food matrix may exert different effects on the bioaccessibility of polyphenols. For example, some authors have shown how coadministration of cow milk, ascorbic acid or citrus juices could increase the bioaccessibility of green tea catechins due to their stabilization and protection from self-oxidation at alkaline pH [22]. During gastric digestion, most of the polyphenols are released: in fact, this is the phase in which the foods are mainly dissolved. The transformation of food into small particles is due to the effect of pepsin, peristaltic movements, as well as low pH value. The latter could affect the stability of polyphenols as it could favor the transition from the food matrix to the aqueous phase thanks to the reduced ionic interactions. At the small intestine level, the pH increases, and different enzymes and biosurfactants that mainly contribute to the digestion of nonpolar compounds such as lipids, nonpolar micronutrients and phytochemicals with the formation of water-soluble micelles, can be found. Generally, polyphenols are stable in the acidic gastric environment, but are degraded in the neutral and weakly alkaline environment of the small intestine. A very important parameter affecting polyphenols’ bioavailability appears to be lipophilicity. Indeed, a direct correlation between lipophilicity and enterocyte permeability has been demonstrated. In the intestine, polyphenols undergo phase I/II metabolism. In enterocytes, excretion of polyphenols towards the intestinal lumen can occur through ATP-binding cassette transporters (ABCs). Other transporters, such as multidrug resistance proteins (MRPs) and monocarboxylate transporters, play a role in the transport of polyphenols to the vascular side [23]. Furthermore, it has been shown that MRP2 and p-glycoprotein play a pivotal role in the efflux of metabolized polyphenols as highlighted in a Caco-2 cell transwell model. Flavonoids that are not absorbed in the small intestine meet the colon microbiota capable of deglycosylating, oxidizing and demethylating the non-absorbed flavonoids, producing small phenolic acids. Conversely, some authors have shown that the glucose position (3′ and 4′) does not affect the absorption rate. This behavior could be explained by the fact that quercetin glucoside transport into enterocytes is mediated by SGLT 1 independently from the sugar moiety position [24]. Many studies indicate that quercetin is an inhibitor of the multidrug resistance protein (MDP). Furthermore, this flavonol inhibits p-glycoprotein by direct binding to this efflux pump or by downregulation of its expression [25]. Absorption of quercetin is influenced not only by the type of sugar and its position, but also by the food matrix. For example, apple peel is rich in quercetin, but high amounts of insoluble fiber may interfere with its intestinal absorption. Rutin, the main flavonol present in apples and tea, is composed of quercetin and disaccharide rutinose characterized by a β-glycosidic bond. It is known that humans do not have the necessary enzyme pattern to break down this type of bond: the bacterial flora present in the colon mediates this hydrolysis, leading to minimal intestinal absorption and the production of phenolic acids as metabolites. Quercetin glucosides are absorbed through SGLT1 which is an active transporter that requires energy for its function and thus leads to a higher absorption rate than passive transport of aglycones through the intestinal wall. Since lipid solubility is a key factor in the absorption of flavonols, it is important to take into account the role of dietary fats in the rate and extent of absorption. In fact, several authors have shown how the intake of aglycone quercetin together with fat foods leads to a higher plasma concentration of quercetin in comparison with the intake of flavonols with low-fat meals. The increased absorption of quercetin when taken together with a high-fat meal can be explained by the fact that a greater incorporation into the micelle and soluble fat droplets is obtained [26]. After absorption, quercetin is transported to the liver where it undergoes phase I and II metabolic reactions, producing metabolites that reach all parts of the body through the blood circulation [27]. Bioavailability of quercetin can be investigated and understood through the identification of its main blood and urinary metabolites. The major metabolites found in blood are quercetin-3-sulfate, -3′-sulfate and -3-glucuronide, while the major 24-h urinary metabolites are quercetin-diglucuronide, -3′-glucuronide, isorhamnetin-glucuronide, -glucuronide sulphate and -methyl quercetin diglucuronide. The kidneys play a pivotal role in the metabolism of quercetin: in fact, renal metabolism includes glucuronide and sulphate conjugates on different sites of quercetin structure. Quercetin metabolites appear in blood 30 min after ingestion and are eliminated in significant amounts in 24 h. This indicates that quercetin has a short plasma half-life and a rapid clearance. It is well-known that black tea contains flavonol kaempferol; however, studies evaluating the bioavailability of food-derived kaempferol conjugates are limited [28]. Some authors have shown that, after taking 27 mg of kaempferol from black tea for three days, the renal excretion of kaempferol was 2.5% of the administered dose, suggesting that absorption of this flavonol is greater than that of quercetin. The greater bioavailability of kaempferol compared to quercetin, despite the latter being more present in tea, is due to the different structural features. As already discussed, flavonols are extensively metabolized in the liver and are found in the systemic circulation in the form of sulfate, methyl and glucuronide conjugates. Identification of the major metabolites present in the circulation after a meal rich in kaempferol is a crucial step in understanding the potential bioactive metabolites. In humans, after a kaempferol-rich meal, the main metabolite found in blood and urine was kaempferol-3-glucuronide [29]; furthermore, small amounts of kaempferol mono- and di-sulfate were detected in urine. Unlike quercetin, which, due to its rapid metabolism, does not resist as an aglycone in blood and urine, kaempferol is found in plasma and urine (40% and 16% of the total kaempferol ingested, respectively). These results indicate that in the human body, there is a high activity of enzyme β-glucuronidase which hydrolyses glucuronides. Furthermore, the lower concentration of kaempferol in urine than in blood indicates that some of the aglycones are metabolized in the kidneys before excretion. Kaempferol is capable of reversing the drug resistance promoted by ABCG2 [30] and inhibiting quercetin efflux by this transporter. ABCG2 and ABCC2 play an active role in the in vivo elimination of kaempferol-3-glucuronide (the main metabolite of kaempferol). Finally, the cooperation between ABC transporters and UDP-glucuronosyltransferases seems to regulate glucuronidation of kaempferol, thus regulating accumulation at the cellular level (in comparison to the glucuronide forms) and bringing effects on the pharmacological properties of this molecule. Fisetin is another important flavonol present in different fruits and vegetables (2–160 μg/g) [31]. It has been shown that after oral administration, fisetin is rapidly metabolized into glucuronide demonstrating that less sulfation occurs in enterocytes than in hepatocytes compared to the i.v. administration. As for distribution, fisetin is concentrated mainly in the kidneys, intestines and liver [31]. The three main metabolites of fisetin are generally referred to as M1, M2 and M3. Metabolites M1 and M2 are glucuronides, while metabolite M3 is a 3′-O-methylated fisetin, namely, geraldol [32]. From the metabolic point of view, in vivo methylation of fisetin could be seen as a favorable path since the formation of the methylated derivative leads to greater stability of flavonol, which could be useful to retain more of the molecule. Furthermore, the presence of the methoxy group on ring A or B seems to protect from bacterial degradation in the feces leading to a longer residence time within the intestines and thus allowing the reabsorption of the intact methylated metabolite from the intestines [31]. Considering that, even if further studies are still necessary to deeper and better understand the pharmacokinetics of flavonols, in vitro and in vivo studies of this class of flavonoids are being conducted continuously and represent a critical step to better understand the results obtained from human clinical studies.

## 3. Health-Promoting Properties and Antioxidant Activity

The focus on the beneficial properties of phenolics appeared in the 1980s, with the seminal work by several research groups that paved the way to the understanding of the many roles they play in disease prevention and, in general, as health-promoting agents. Following the pioneering findings by Cody et al., Hertog et al. as well as Rice-Evans et al. turned their attention to the action of flavonoids as radical scavengers and on the structural features of these ubiquitous compounds responsible for their antioxidant activity [9,33,34]. Fueled by these early reports—as well as by consumers’ attention to the unquestionable beneficial effects of eating flavonoid-containing fruits and vegetables—a huge number of reports has been published on the isolation, identification and quantification of subclasses of flavonoids from natural sources, on their many properties and on their structure–activity correlations. Among the flavonoids, flavonols have shown, in general, a stronger activity than the other members of the flavonoid family. In vitro and in vivo studies as well as human clinical trials provided clear evidence that these bioactive compounds (and/or their metabolites) do not merely possess an antioxidant activity, but the spectrum of their action spans from the prevention of several chronic and cardiovascular diseases to metabolic disorders. To date, few human clinical trials have been carried out to investigate effects of dietary intake of flavonols. The evidence presented on the properties of flavonols is discussed in the following sections. The antioxidant activity of flavonols is the property (more than any other) that makes these compounds extremely attractive [35]. This ability can be exploited in many ways, both in in vivo applications as well as in biotechnological ones. From the biological point of view, flavonols have an outstanding ability to protect from oxidative stress, thus limiting oxidative damage and reducing the severity of chronic diseases. The ability to scavenge free radicals also makes it possible to employ flavonols as food preservatives, increasing shelf life and stability of fresh food products [36]. Moreover, besides free radical quenching, these compounds are able to regenerate antioxidant vitamins C and E [37].

Flavonols are able to quench radical species by following different mechanisms, acting both via hydrogen atom transfer (HAT) or electron transfer (ET) [38]. In addition, phenolic OH groups may undergo deprotonation, thus reacting with free radicals at a faster rate according to a sequential proton loss electron transfer (SPL-ET) mechanism [39]. The preferred radical quenching mechanism ultimately depends on the extent of flavonol deprotonation, although these mechanisms often take place simultaneously during free radical scavenging.

**Figure 1:**
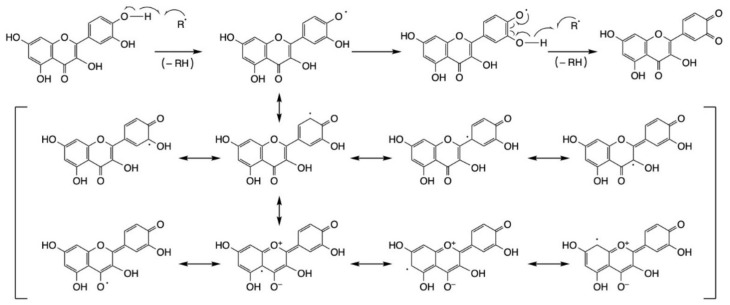
Free radical (R•) quenching mechanism for quercetin, along with the most relevant resonance structures for the intermediate aryloxy radical formed upon reaction with the first R• species.

Taking all of the above into account, a number of different radical scavenging assays, based on both hydrogen atom transfer and electron transfer-based methods, have been used to assess and quantify the antioxidant ability of flavonols [40]. Most commonly, DPPH, ORAC, TEAC and FRAP assays have been employed, although the response obtained for individual flavanols may vary depending on variables such as the mechanism of radical quenching, the charge of the radical (positive, negative or neutral), the polarity of the solvent used, the solubility of the tested molecules or the pattern of glycosylation of the tested flavonols (Figure 1).

Antioxidant data from recent literature has recently been reviewed [41]. Some structure–activity correlations can be easily drawn: flavonols possess many structural features that make the best of the other flavonoid subclasses in terms of radical quenching. In fact, the presence of the 2,3-double bond on ring C, similarly to flavones, dramatically increases conjugation between the aromatic ring B and the other aromatic portion of the molecule, the benzoyl moiety composed of ring A and the carbonyl group. Higher conjugation results in a likewise higher stability of the radical formed upon free radical scavenging, thus making flavonols’ antioxidant action extremely efficient.

A further structural feature that makes some flavonols particularly efficient is the presence of two OH groups on the B ring in a catechol-type arrangement (i.e., ortho). It is well-known that catechol moieties can react with radicals to give very stable radical products and then, losing two H•, quinone functions [42]. In fact, the importance of the catechol ring is demonstrated by the comparison between the ability of quercetin and isorhamnetin to quench the DPPH radical (6.5 ± 1.90 vs 126.48 ± 4.26 μM). Furthermore, the hydroxyl group in the 3-position of ring C plays a fundamental role in radical scavenging. In fact, glycosylation at the 3-position results in a significant decrease in DPPH quenching ability [43], with quercetin-3-O-rhamnoside and quercetin-3-O-galactoside returning 47.68 ± 1.60 and 53.34 ± 2.64 μM, respectively, at the DPPH test. Figure 1 depicts a schematic representation of the scavenging activity of quercetin following interaction with radicals. Interestingly, in the inhibition of lipid peroxidation, isorhamnetin and tamarixetin (i.e., methylated metabolites of quercetin) performed better than quercetin itself [44]. However, both the catechol moiety and the 3-OH group do not have an influence on the ORAC assay, with all the compounds mentioned above scoring in the 2.5–5.5 μM Trolox equivalent range [34,45,46,47,48]. Of note are also the antimicrobial and antiviral activity of flavonols. Due to the increased resistance to antimicrobial and antiviral drugs, more effort is currently focused on the identification of novel therapeutics to be used alone or in combination with the existing compounds.

Several reports indicate an antimicrobial and antiviral effect of plant extracts [49,50,51]. We have demonstrated the antimicrobial and antiviral potential of pistachio extracts containing flavonols, including kaempferol-3-O-rutinoside, quercetin, quercetin-3-O-rutinoside and quercetin-3-O-glucoside, against standard and clinical strains of *Staphylococcus aureus* and herpes simplex virus 1 (HSV-1), respectively [52,53]. The antimicrobial, antifungal and antiviral potential of flavonols and their mechanisms of action were discussed in our recent paper [41]. It is worth exploring their potential synergistic effect in combination with traditional compounds: a recent investigation indicated that the interaction between quercetin and naringenin dictates the biological effect of the mixture [54]. Nanoencapsulation of brain-boosting polyphenols including quercetin, caffeine and cocoa flavanols could provide new insights to address the COVID pandemic [55]. The synergistic effect of quercetin with antibiotics against multidrug-resistant clinical strains of *Pseudomonas aeruginosa* has also been reported [56]. Flavonol isorhamnetin combined with ferulic and gallic acid reduced cell viability when bactericidal and fungicidal activities were evaluated [57]. The combination of kaempferol with aminoglycosides may be effective in the treatment against *S. aureus* and *E. coli* due to their synergistic association, reducing the MIC values and thus decreasing the dose for a therapeutic approach. Taken together, these data indicate an antimicrobial and antiviral effect of food flavonols. These remarkable activities can be attributable to different elements, and the great capacity of charge distribution of these molecules is to be attributed to the multiple tautomeric forms, these characteristics common with other carbonyl and hydroxyl aromatic molecules [58] that justify their fundamental biological role. More effort is needed to understand their mechanisms of action, especially when associated with other drugs.

## 4. Biotechnological and Industrial Applications of Flavonols

**Figure 2:**
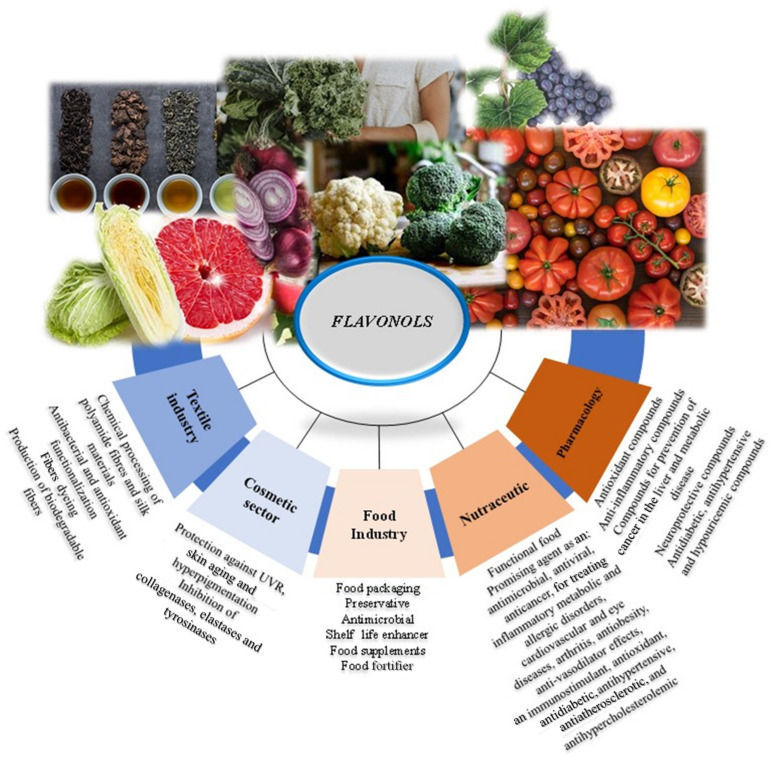
Schematic representation of the main biotechnological applications of flavonols.

Flavonols are bioactive compounds widely used in several applications thanks to their broad spectrum of properties, from the food, nutraceutical, pharmaceutical, medicinal and cosmetic sectors to the textile industry (Figure 2). Several studies have highlighted the functional properties of flavonols such as the anti-inflammatory, antioxidant, antiviral, antimicrobial, anticancer ones and their absorption capability [41,59,60,61,62,63,64]. In addition, many industrial byproducts represent safe and inexpensive bioactive sources of flavonols which, as natural bioactive molecules, could be exploited in different applications, such as the nutraceutical and cosmetic industry and mainly as additives and/or functional ingredients for the fast-growing functional food industry [65]. A list of the biotechnological properties and bioactivity of flavonols are depicted in Table 2.

### 4.1. Flavonols and Food Industry

In the last years, the interest in innovative technology, by using natural compounds which could boost the shelf life and the microbial safety of food products, has greatly increased [66].

Flavonols may play a functional role in several processing steps of different foods, from production to packaging. Phenolic extracts, including flavonol compounds such as glycosides of quercetin and kaempferol or myricetin, kaempferol and quercetin, have been shown to constitute interesting tools as natural preservatives of several food products such as minced pork meat [67,68] and rice beer [69]. Given the functional properties of flavonols, nowadays, scientific interest is focused on obtaining these bioactive molecules from plants or from agroindustrial byproducts for their use as food supplements in the production of functional foods and beverages. The use of innovative extraction methods, such as supercritical fluid technology and stabilization of these molecules, has been investigated, and innovative applications in the food industry have been promoted [70].

The use of quercetin during soy sauce heat treatment significantly decreases ethyl carbamate formation. Zhou et al. showed that optimized contents of quercetin together with ornithine promote a reduction of the ethyl carbamate concentration in soy sauce during heat treatment with minimal effects on the color and volatile flavor compounds of soy sauce [71].

The interaction of carbohydrates and flavonols improves the physicochemical properties of starch, promoting several applications in the food industry. Flavonols can change properties such as retrogradation, gelation, gelatinization and digestibility of starch. In particular, quercetin and rutin are capable of delaying retrogradation, reducing the gelatinization temperature and facilitating formation of a softer starch gel [72]. Quercetin together with starch has been used to produce a new resistant starch with a strong antioxidant potential, useful as an encapsulation compound and packaging material [63]. In addition, quercetin, used as a preservative together with 4-hexylresorcinol and cinnamic acid, in combination with atmosphere packaging has been found effective in reducing the risk of disorders caused by biogenic amines in Pacific white shrimp [73]. The antimicrobial effectiveness of quercetin has been proven, and its possible use as an alternative antibiotic feed additive agent in animal production has been highlighted [74]. Furthermore, this molecule showed no activity on *Lactobacillus casei* var shirota, resulting in it being very interesting as an additive in functional foods [75]. Several studies reported that the consumption of flavonol-rich foods can change the gut microbiota composition, resulting in a prebiotic-like effect [76,77].

Quercetin has been shown to be an interesting compound in the food packaging field as well. Recently, quercetin has been used as an active ingredient in poly(vinyl alcohol) (PVA) film formulations, can be used to improve the antioxidant properties and the deformability of the produced films [78]. A recent study revealed that β-cyclodextrin/quercetin inclusion complex nanofilms had a high inhibitory effect on *S. aureus* and *E. coli* and, thanks to the timed release of quercetin from the inclusion complex nanofiber membrane, the quercetin active time could be prolonged [79]. The complexation of quercetin with methyl-β-cyclodextrin slightly reduces its photodegradation without significantly limiting its antiradical, antioxidant and metal-chelating properties [79].

Morin has been shown to be employable in antibacterial and antioxidant treatments [80] and in promoting the delay of the banana skin color change from green to yellow, the slowdown of senescence and inhibition of fungal infection [81].

Myricetin has also been widely applied in the food industry as a preservative agent to prolong the shelf life of foods rich in oils and fats thanks to its ability to protect lipids against oxidation [82].

### 4.2. Flavonols and Nutraceutical and Pharmacological Field

Thanks to their high nutritional and medicinal value, flavonols are considered important nutraceuticals; several studies have been conducted in order to look into their wide range of health benefits and pharmacological applications [41,83,84]. Flavonols are the most prominent flavonoids in fruits and vegetables and, among these compounds, quercetin is the most frequently consumed in the human diet [85].

Quercetin is one of the most important plant compounds; it has been used as a nutritional supplement [85] and can be found as a food supplement in the powder and capsule forms [27]. It may be beneficial against a variety of diseases given its several pharmacological activities, such as being antimicrobial, antiviral, anticancer, for treating inflammatory metabolic and allergic disorders, cardiovascular and eye diseases, arthritis, anti-obesity, anti-vasodilator effects, an immunostimulant, antioxidant, antidiabetic, antihypertensive, antiatherosclerotic and antihypercholesterolemic [27,47,85,86,87,88].

However, its pharmaceutical application is restricted by its poor absorption into the body due to its instability, poor solubility, poor permeability and low bioavailability.

In order to increase its application, structural modification with glucoside–sulfate conjugates, complexes with metal ions, such as vanadium, cadmium, magnesium, calcium, cobalt, copper, ruthenium and iron, or with ionic complexes, such as calcium phosphate–quercetin nanocomplexes, glucan–quercetin conjugates and quercetin–germanium nanoparticles have been shown to improve quercetin bioavailability and increase its antioxidant activity and scavenging capacity. In addition, innovative quercetin preparations comprising quercetin-loaded mucoadhesive nanoemulsion, quercetin-loaded gel, quercetin-loaded polymeric micelle and quercetin-loaded nanoparticles have been developed to enhance the bioavailability and solubility of quercetin, which not only improve its clinical efficacy but also present novel drug preparations for research and development [86].

Kaempferol also found application in the treatment of numerous diseases. It has been shown to have beneficial effects on health, mostly for its well-known anti-inflammatory effects, followed by its significant role in cancer prevention and in liver and metabolic disease [59]. However, in order to overcome its poor bioavailability and improve its pharmacokinetics, the use of kaempferol nanosuspensions [89], solid dispersions [90] and complexes of polysaccharides and oligosaccharides [88] has been developed.

Morin exerts antioxidant activity, but also anti-inflammatory, antidiabetic, antihypertensive, antitumoral, antibacterial, neuroprotective and hypouricemic effects by modulating the activity of numerous enzymes. In addition, in some cases, morin or its water-soluble derivative, morin-5′-sulfonic acid sodium salt, showed protective action, reducing negative side effects of various drugs without interfering with their effects [91,92].

Myricetin shows various pharmacological activities including antioxidant, anti-inflammatory, iron-chelating and anticancer properties [61,82]. Recently, efficient formulations for the delivery of myricetin have been developed in order to increase the dissolution rate and aqueous solubility of myricetin or to prevent rapid degradation in high-pH intestinal fluids such as self-nanoemulsifying drug delivery systems [93], polyvinyl caprolactam–polyvinyl acetate–polyethylene glycol graft copolymer polymeric micelles [94] and myricetin-mediated gold nanoparticles [95]. Fisetin is another well-known flavonol that has excellent anti-Parkinson’s, anticancer and antioxidant activity. Nutritional supplements exhibited several pharmacological effects, as well as anti-inflammatory and antioxidant activity [96]. Novel water-soluble fisetin/β- and γ-cyclodextrin complexes and polymeric micelles have been developed for medical applications and food industry [97,98].

### 4.3. Flavonols and Cosmetics

Flavonols have recently attracted considerable interest for their ability to confer defense against UV radiation-induced skin damage through both direct photoabsorption and antioxidant activity [99] and prevent skin aging and hyperpigmentation caused by solar UV irradiation [100].

In particular, Maini et al., using an artificial skin mimic, showed that quercetin, kaempferol and galangin applied topically may protect skin from UVR-induced damage. Considering that the more stable flavonols galangin and kaempferol may be the most promising topical skin photoprotectants, they hypothesized that the ability of flavonols to protect against UVR-induced damage would be inversely proportional to hydroxyl group substitution on the B ring [101].

In addition, a new acylated kaempferol glycoside with a rare structure, kaempferol 3-O-β4C1-(6”-O-3,4-dihydroxyphenylacetyl glucopyranoside), which has been isolated from *Prunus persica* (L.), kaempferol, quercetin and myricetin were reported to have anti-elastase and anti-collagenase activity [102,103,104]. Kaempferol and quercetin also have an inhibitory effect on tyrosinases [100]. Inhibiting the three enzymes, collagenases, elastases and tyrosinases, which are known to play a crucial role in skin aging, increases the strength of the skin, improves elasticity and helps to avoid development of dark spots, preventing the formation of wrinkles [102]. Thanks to the C-3-hydroxyl group, flavonols are stronger inhibitors of collagenase than flavones and isoflavones [102]. Myricetin could also be used in cosmetic preparations given its protective effects against skin aging [82].

In addition, several encapsulation techniques have been tested in order to stabilize these natural compounds during storage and increase bioavailability and/or skin permeability in cosmetic preparations, enhancing in some cases dermocosmetic performance with topical application. Several micro- and nanocarriers including liposomes, microspheres, nanoparticles and nanoemulsions have been found to be promising tools to overcome the quercetin limitations due to low solubility, stability and skin permeability [105].

### 4.4. Flavanols and Textile Industry

Recently, one of the fields in which flavonols represent increasingly promising alternatives is the treatment of fibers in the textile industry, mostly thanks to their biocompatibility, low irritability and eco-friendliness [60,64,106,107].

Quercetin has been shown to be one of the most promising multifunctional agents for the chemical processing of polyamide fibers and silk materials using an absorption technology (Table 2).

Zhou and Tang demonstrated that rutin and quercetin can be used as multifunctional agents for the chemical processing of silk materials. Although quercetin has been shown not only to exhibit a higher initial absorption capability than rutin thanks to its good affinity constant, but also to impart better UV protection, antibacterial and antioxidant performance to silk and provide better washing durability [64]. Quercetin and rutin also promote a simultaneous antibacterial and antioxidant functionalization of polyamide fibers. Quercetin, employed in the treatment of polyamide fibers, exhibited not only a higher affinity and absorption capability, but also higher antioxidant and antibacterial activity than rutin. In addition, these functions displayed good resistance to washing [60].

Furthermore, flavonols have been tested for fiber dyeing in textile processing, promoting environmentally friendly processes [106,107,108]. In particular, the possible application of quercetin, morin and rutin in dyeing cotton, using an enzymatic process catalyzed by laccases, could avoid bleaching pretreatment and maintain the naturally occurring flavonoids on cotton, which play a significant role in the grafting reaction, enhancing color fastness and dyeing [106,107]. Furthermore, quercetin, compared to morin, showed acceptable values of color strength for the coloration of flax fabrics [107] and, under ionizing radiation, showed that it can be used safely as a possible alternative to reddish-brown synthetic dyes [109].

Another application of flavonols in this field consists in the production of biodegradable fibers, which are effective against several microorganisms, particularly those with antibiotic resistance. In this context, quercetin (Q)-loaded polylactide-based fibers developed using the electrospinning technique appeared to be more effective than those containing antibiotics, representing a promising instrument for obtaining efficient and stable antibacterial dressing materials [110].

**Table 2:** Most common utilization of flavonols.

Flavonol	Bioactivity and Biotechnological Applications	Application	References
Flavonols and food industry			
Quercetin, kaempferol, myricetin, isorhamnetin Quercetin and kaempferol glycosides Morin	Antimicrobial and antioxidant activity	Food preservatives	[67,68,69,73,75,80,81,82]
Kaempferol, quercetin, kaempferol and quercetin glycosides	Antioxidant, anticancer, anti-inflammatory, hepatoprotective, neuroprotective, cardioprotective properties, antibacterial and prebiotic-like activity	Production of functional food products and food supplements	[27,70,75,76,77]
Quercetin, rutin	Ethyl carbamate reduction in soy sauce Physicochemical properties improvement	Food fortifier	[63,71,72]
Quercetin	Antioxidant, antiradical, metal-chelating, anti-lipoperoxidative, antimicrobial activity	Food packaging	[63,72,73,78,79]
Quercetin	Antimicrobial activity	Alternative antibiotic feed additive in animal production	74
Flavonols and nutraceutical and pharmacological field			
Quercetin, rutin	Antimicrobial, antiviral, anticancer, anti-obesity, anti-vasodilator effects, immunostimulant, antioxidant, anti-diabetic, antihypertensive, antiatherosclerotic and antihypercholesterolemic activity, treatment of inflammatory metabolic and allergic disorders, cardiovascular and eye diseases, arthritis	Nutritional supplement and innovative complexes and formulations for pharmaceutical application	[27,47,85,86,87,105]
Kaempferol	Anti-inflammatory activity, cancer and liver and metabolic disease prevention	Innovative complexes and formulations for pharmaceutical application	[59,70,88,89,90]
Morin	Antioxidant activity, anti-inflammatory, antidiabetic, antihypertensive, antitumoral, antibacterial, neuroprotective, hypouricemic, enzyme-modulating	Pharmaceutical application	[91,92]
Myricetin	Antioxidant, anti-inflammatory, iron-chelating, anticancer properties	Innovative complexes and formulations for pharmaceutical application, increase the bioavailability of others	[61,82,93,94,95]
Fisetin	Anti-Parkinson’s, anticancer, anti-inflammatory and antioxidant properties	Pharmaceutical application Innovative complexes and formulations for pharmaceutical application	[96,97,98]
Flavonols and cosmetics			
Quercetin, kaempferol, galangin	Protection against UVR Anti-inflammatory and antioxidant effects	Skin photoprotectants Dermocosmetic application	[99,100,101,105]
Kaempferol, quercetin	Inhibition of tyrosinases	Whitening agent in skin disorders	[100]
Kaempferol 3-O-β4c1-(6”-O-3,4-dihydroxyphenylacetyl glucopyranoside), kaempferol, quercetin, myricetin	Inhibition of elastases and collagenases	Innovative complexes and formulations for antiaging application	[82,102,103,104]
Flavanols and textile industry			
Quercetin, rutin	Absorption capability UV protection, antibacterial and antioxidant activity	Functionalization of polyamide and silk fibers	[60,64]
Quercetin, morin, rutin	Dye sources	Textile dyeing	[106,107,108,109]
Quercetin	Antimicrobial activity	Antibacterial dressing materials Production of biodegradable fibers	[110]

## 5. Evidence from Intervention Clinical Trials

Plants provide a wide range of secondary metabolites such as polyphenols, which are responsible for the inverse correlation between fruit and vegetable intake and onset of chronic degenerative diseases. Among them, flavonoids represent the most abundant class widespread in plant-based food and beverages, consisting of more than 5000 members [111]. Thanks to their well-documented antioxidant and anti-inflammatory activity, these compounds exert, through different signal pathways, different health benefits [111].

Potential cardioprotective effects have also been attributed to kaempferol. However, data on dietary kaempferol bioavailability and absorption in humans are limited, and no clinical trials are currently available on the cardiovascular effects. The reviewed studies indicate that a daily intake of kaempferol ≥ 1.5 mg/day is associated with a lower incidence of coronary heart disease and myocardial infarction, but the limited data available do not allow establishing a positive correlation between kaempferol intake and cardioprotective effects [28].

The therapeutic potential of quercetin and rutin has been widely analyzed, especially as far as antiproliferative and antioxidant effects for many diseases are concerned, including the neurological pathologies, although the neuroprotective effects of this subclass of compounds in human studies has been poorly tested [112]. Recently, a systematic review has suggested that quercetin could be an interesting preventive strategy to counteract hormonal disturbances and the subsequent metabolic disorders which occur in polycystic ovary syndrome (PCOS) [113]. Evidence suggests that quercetin improves the ovarian histomorphological scenario similarly to metformin, improving the serum levels of testosterone and luteinizing hormone (LH), through decreasing the resistin levels. It is well-known, indeed, that PCOS-induced hyperandrogenemia as well as chronic inflammation and oxidative stress may lead to weight gain and insulin resistance, leading to anovulation [113]. However, in this case, none of the included studies recorded the blood concentration of quercetin to determine its bioavailability; as a consequence, the optimum dose of quercetin necessary to reduce the amount of metformin has not been found, and further well-designed studies with longer duration are necessary to reach conclusive results about the positive effects of quercetin supplementation in women affected by PCOS [113].

These results are in accordance with a previous clinical study, which showed a significant decrease in the IL-8 levels without any modification of IL-10 after LPS-induced inflammation. Moreover, even though there have been no RCTs available on fisetin or related flavonols in cancer to compare the findings of the above study regarding MMPs, it was already demonstrated in healthy male subjects that quercetin supplementation for two weeks had not resulted in any significant alterations in MMP-2 gene transcription or plasma protein levels.

Starting from this evidence, fisetin may represent a starting point for further studies as a promising compound in cancer prevention and therapy [114].

## 6. Synthetic and Semisynthetic Flavonols and Flavonoid–Metal Ion Complexes

Flavonoid modification by chemical means—or even their total synthesis—is an approach that provides access to a range of promising semisynthetic or synthetic bioactive species that can lead, eventually, to new drugs capable of overcoming the drug resistance developed by many microorganisms. Peripheral modifications to the OH groups or to the glycosyl moieties have been carried out (oxidation, halogenation [115], glycosylation [116]), as well as more profound modifications to the phenyl benzopyranone core by replacing its all-carbon aromatic rings with heteroaromatic units, such as pyridine, piperidine or even 1,3-dithiolium cations [50].

All the chemistry operations carried out on these compounds have led to a pool of derivatives displaying a wide diversity, making it difficult to draw any definitive conclusions regarding their structure–activity relationship. However, a lot of effort has been invested in this direction, and reviews have recently been published on the potential activity of synthetic flavonoids against cancer cells or specific pathogens. For instance, a very in-depth survey examined the state of the art of the potential treatment of leukemia with synthetic flavonoids [117]. The structure–activity relationship was analyzed in an attempt to correlate the different mechanisms by which they act, such as inhibition of cell growth and proliferation by arresting the cell cycle or induction of apoptosis and differentiation.

Very recently, in silico screening of 101 synthetic derivatives of natural flavonoids showed that thioflavonols (i.e., with a 4-pyranthione ring C) are highly promising inhibitors of the main protease (M^pro^) of SARS CoV-2, a protease that plays a central role in the lifecycle of the virus [118].

Part of the research on the therapeutic potential of flavonols regards their ability to complex metal ions. Chelates provide new potential drugs with a broader spectrum of pharmacological activities, as well as enhanced antioxidant and anticancer activities, with flavonols performing better than flavanones or flavones [119]. To the best of our knowledge, there has been no in-depth study on the structure–activity relationships of flavonoid complexes of metal ions; nonetheless, possible mechanisms of action have been proposed, mostly relying of DNA binding and apoptosis induction [120]. Moreover, because of disadvantageous physicochemical properties of 3-hydroxyflavones, such as low photostability and poor water solubility, the inclusion complex between 3-hydroxyflavones and methyl-β-cyclodextrin has been shown to be a potential active flavonoid carrier [121].

## 7. Conclusions

The unquestionable biological properties of flavonols, as well as their biotechnological and health-promoting properties, offers the possibility to produce functional foods and beverages that may display not only an enhancement of their functional chemistry, but also an increase in their stability and shelf life. Moreover, the increased consumers’ attention to these molecules results in a remarkable progress and development of their utilization due to the lack or insignificant adverse effects or toxicity associated with their metabolization and, on the other hand, their health-promoting properties. The optimization of extraction techniques and the use of green solvents, along with the development of new downstream processes of production, can further promote the development of flavonol utilization. In particular, the biotechnological potential of these molecules makes them a candidate of choice for a variety of purposes, from pharmaceutical/cosmetical applications to fast-growing functional food and textile industries, also supported by the possibility of green utilization of several industrial byproducts, that are a rich and inexpensive source of flavonols. Taking into account that every year tons of materials are produced as residues by the agri-food industry, they can become a resource and add value to the sector exactly due to the presence of these secondary metabolites. Moreover, the presence of the hydroxyl group at C-3 makes them strong enzyme inhibitors in cosmetic preparations, such as myricetin. Another, almost completely forgotten aspect of these molecules, is the possibility to produce probiotics (as products of human gut microbiota or by direct fermentation before intake) to increase bioavailability of other products. Quercetin, in particular, is capable of improving gut dysbiosis after antibiotic treatment and finely modulate the gut microbiota composition. On the whole, the data presented in this review support the new perspectives for future basic and translational studies within this nascent and promising research field that may help to substantially increase flavonol utilization.

## Figures and Tables

**Table 1:**
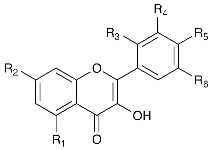
Most common flavonol aglycons.

	R_1_	R_2_	R_3_	R_4_	R_5_	R_6_
Quercetin	OH	OH	H	OH	OH	H
Kaempferol	OH	OH	H	H	OH	H
Myricetin	OH	OH	H	OH	OH	OH
Isorhamnetin	OH	OH	H	OCH_3_	OH	H
Fisetin	H	OH	H	OH	OH	H
Galangin	OH	OH	H	H	H	H
Rhamnetin	OH	OCH_3_	H	OH	OH	H
Morin	OH	OH	OH	H	OH	H

## Data Availability

Not applicable.
